# A comparative study on fundamental movement skills among children with autism spectrum disorder and typically developing children aged 7–10

**DOI:** 10.3389/fpsyg.2024.1287752

**Published:** 2024-03-28

**Authors:** Liangshan Dong, Rong Fan, Bo Shen, Jin Bo, Yanli Pang, Yu Song

**Affiliations:** ^1^School of Physical Education, China University of Geoscience, Wuhan, China; ^2^Division of Kinesiology, Wayne State University, Detroit, MI, United States; ^3^Department of Psychology, Eastern Michigan University, Ypsilanti, MI, United States; ^4^School of Physical Education, Central China Normal University, Wuhan, China; ^5^School of Physical Education, Ji Mei University, Xiamen, China

**Keywords:** children with autism spectrum disorders, typically developing children, fundamental motor skills, motor deficits, MABC-2

## Abstract

**Background:**

Autism Spectrum Disorder (ASD) is a neurodevelopmental condition with unique differences in social interaction, communication, and a spectrum of behavioral characteristics. In the past, motor disturbance in individuals with ASD has not been considered a significant core deficit due to the predominant focus on sociability and communication issues. However, recent studies indicate that motor deficits are indeed associated with the fundamental symptoms of ASD. As there is limited research on the motor behavior of children with ASD, particularly in China, the objective of this study is to investigate the development of fundamental movement skills (FMS) in children with ASD and compare them to typically developing children.

**Method:**

The study recruited 108 children with ASD (87 boys, 21 girls) aged 7–10 years from two special education rehabilitation centers in Wuhan, China. For comparison, a control group of 108 typically developing children, matched by age and gender, was randomly selected from three local primary schools. FMS were assessed using the Movement Assessment Battery for Children - Second Edition (MABC-2), which evaluates manual dexterity, aiming and catching, as well as static and dynamic balance. Group differences on MABC-2 percentile scores were analyzed using descriptive statistics and Mann–Whitney U test. Effect sizes were also calculated for practical significance.

**Results:**

Findings from the study showed that a significant majority, around 80%, of children with ASD either displayed motor challenges or were at risk of developing such delays. When comparing to their typically developing peers, children with ASD scored notably lower in areas of manual dexterity, ball skills, and both static and dynamic balance (with all these findings being statistically significant at *p* < 0.001). Interestingly, gender did not show a significant influence on these results (*p* > 0.05).

**Conclusion:**

In addition to addressing the other skill development areas outlined in the diagnostic manual for ASD, clinicians diagnosing and treating children with ASD should also assess the presence of motor skill development. For individuals with ASD who have co-existing motor difficulties, it is essential to offer evidence-based interventions tailored to their specific needs.

## 1 Introduction

Autism Spectrum Disorder (ASD) is a neurodevelopmental condition with unique differences in social interaction, communication, and a spectrum of behavioral characteristics (American Psychiatric Association, 2013). While motor skills are not a diagnostic criterion for ASD, an increasing body of research suggests that children with ASD commonly experience significant motor development difficulties, to the extent that some researchers have discussed including motor impairments as part of the diagnostic criteria (Lane et al., 2012; Liu, 2012; Whyatt and Craig, 2012; Liu and Breslin, 2013; Miller et al., 2021; Bhat, 2022; Licari et al., 2022). Early detection and targeted intervention have been emphasized as crucial for the effective treatment of ASD (National Research Council, 2001; Corsello, 2005).

Recent research underscores the critical nature of motor impairments in children with ASD, positioning these challenges as potential early markers of the disorder. A systematic review by highlights that children with ASD face significant difficulties in fundamental movement skills (FMS), such as object control and locomotor skills, when compared to their typically developing counterparts. This finding emphasizes the importance of incorporating FMS assessments into the routine evaluations for children with ASD to identify and address these impairments early. Complementing this, Kaba et al. (2022) demonstrated that movement-based interventions could substantially enhance imitation and praxis skills among this population, advocating for the early initiation of targeted interventions to mitigate motor skill deficits.

FMS are pivotal for enabling participation in a wide array of activities, from casual play to structured sports, serving as foundational elements for the acquisition of more complex skills (Hands, 2012). Furthermore, motor skills play a crucial role in children’s exploration of the environment and participation in social interaction and communication (Wolpert et al., 2003; Bar-Haim and Bart, 2006). Recent research has shown that developmental difficulties in motor skills are fundamental symptoms of ASD and closely related to the core symptoms of ASD, such as social and communication impairments (Haywood and Getchell, 2019). The development of motor skills can regulate the relationship between cognitive and social skills, as it provides a real-life environment and opportunities for children to practice social and communication skills while refining their fine and gross motor skills (Schmidt et al., 2011; Lloyd et al., 2013).

For children with ASD, delays in fine motor development may adversely affect abilities in writing or keyboard input, leading to communication difficulties and limitations in everyday communication. Delays in gross motor development may negatively impact participation in balance and social play activities (e.g., engaging in ball games during recess). Both types of motor skill developmental difficulties can potentially affect the frequency of challenging behaviors in children, making them more prone to avoidance behaviors, such as temper tantrums (Fittipaldi-Wert and Mowling, 2009). Additionally, research has found that early development of motor skills can predict future participation in physical activities. Deficits in motor skills not only reduce opportunities for physical activity and movement in children with ASD but also increase the risk of sedentary-related diseases (Stodden et al., 2008). Untreated motor development difficulties can persist into adolescence and adulthood, leading to long-term physical, psychological, and behavioral issues (Stodden et al., 2008; Davidovitch et al., 2015). Therefore, investigating the characteristics of FMS in children with ASD is of great significance in promoting their levels of physical activity, reducing health risks, and providing early intervention and rehabilitation.

Research into the motor skill development of children with ASD has been rigorously pursued in Western contexts. Yet, the prevailing literature is often marked by constraints such as limited sample sizes, narrow age ranges, and the variability of assessment methodologies (Liu and Breslin, 2013; Pang et al., 2018a,b), casting doubts on the universality of these studies’ outcomes. Additionally, cultural variations in parenting practices may influence the development of motor skills, and these differences need to be considered in research. In response to the limited research on ASD in the Chinese context, this study aims to investigate the development of FMS in Chinese children with ASD aged 7–10 years, using standardized assessment tools. By comparing the development of FMS in children with ASD to that of their typically developing peers, this study aims to enhance our understanding of ASD and facilitate the creation of customized rehabilitation and educational interventions. Focusing on the identification of distinct developmental trajectories and challenges in motor skills among children with ASD, the objective is to inform the design of personalized therapeutic and educational programs in China.

## 2 Methods

### 2.1 Participant demographics and selection

This research was designed to compare two distinct groups: children diagnosed with ASD and their typically developing counterparts, serving as a control group. Each group comprised 108 participants, with an age mean of 7.48 years. The composition was predominantly male, with 87 boys and 21 girls. This gender distribution reflects the well-documented gender disparity in ASD diagnoses, where males are diagnosed more frequently than females (Maenner, 2020).

#### 2.1.1 Group of children with ASD

Participants within the ASD group were sourced from two specialized institutions: Linjie Autism Rehabilitation Center and the Special Children Learning Ability Training Center, both located in Wuhan. The recruitment process was executed in six stages, initially identifying over 150 candidates with ASD for preliminary evaluation. Of these, 108 (87 males and 21 females) successfully completed the requisite assessments. The age distribution was varied, including 39 children aged 7, 30 children aged 8, 23 children aged 9, and 16 children aged 10, ensuring a broad representation across early developmental stages.

Each child in this group had a prior clinical diagnosis of ASD, which was rigorously confirmed by our research team. Additionally, all participants were classified under level 1 of the ASD severity scale as per DSM-5 criteria, indicating mild symptoms necessitating support. To further validate these diagnoses for the purposes of this study, the Autism Diagnostic Interview-Revised (ADI-R) was administered by two doctoral students (Lord et al., 1994). These students had previously undergone comprehensive training and were certified in ADI-R administration by the American Psychological Association (APA), ensuring the assessments’ credibility and reliability.

Given the challenges of assessing IQ through traditional methods in children with ASD, our study employed the C-PEP3 for a comprehensive evaluation. The C-PEP3 assessments indicated that the majority of participants scored within the typical range for cognitive functions, albeit slightly below the normative data for typically developing peers. This tool facilitated a holistic examination across various domains crucial for understanding ASD children’s capabilities. Parental feedback, collected through structured interviews, supported the C-PEP3 results, offering additional insights into everyday adaptive behaviors and cognitive functioning.

These findings underscore the importance of employing specialized assessments like the C-PEP3, designed to accurately reflect the diverse abilities of children with ASD and informing future interventions aimed at enhancing adaptive skills and cognitive functions. This methodological approach allows our findings to accurately depict ASD’s nuanced symptoms, enhancing our understanding of the disorder.

#### 2.1.2 Group of typically developing children

The control group comprised typically developing children, selected from various educational institutions across Wuhan, including three primary schools and one kindergarten. This group was carefully assembled to mirror the ASD group in terms of age and gender distribution, facilitating a balanced and meaningful comparison between the two cohorts. Inclusion criteria for this group involved a screening process using the Social Communication Questionnaire (SCQ) with a cutoff score of 15 to exclude any undiagnosed cases of ASD, complemented by confirmations of typical development from educators and parents. Additionally, the screening process was reinforced by specific screening and entrance standards applied within the Chinese educational system, including academic curriculum tests and standard tests at enrollment, as well as a mandatory health checkup to ensure no significant illnesses that affect normal learning.

For both the ASD and typically developing children groups, exclusion criteria included any developmental disorders such as brain injury or intellectual disability. Before enrolling any participant, consent was obtained from relevant authorities, schools, and parent committees, with parents signing informed consent forms. The study received approval from the local University Institutional Review Board, adhering to stringent ethical standards. To ensure the integrity and ethical conduct of the research, all assessments were conducted with participants’ voluntary cooperation.

### 2.2 Instrument

#### 2.2.1 Movement assessment battery for children-2

The assessment of FMS utilized the MABC-2 (Henderson et al., 2007). The MABC-2 is currently the most widely used standardized assessment tool for assessing motor development in children worldwide and is referred to as the “gold standard test” for evaluating children’s motor development abilities (Green et al., 2011). The MABC-2 comprehensively measures gross motor skills, fine motor skills, and balance abilities of children aged 3–16 years. The test is divided into three age ranges: 3–6 years, 7–10 years, and 11–16 years. This study utilized the measurement standards for the 7–10 year age group.

Each age range assessment consists of three components: manual dexterity, aiming and catching, and static and dynamic balance. There are a total of eight test tasks, and the raw scores of each task can be converted into standard scores. The standard total score (ranging from 1 to 19) can be calculated by summing the standard scores of the eight tasks. Based on the normative conversion table in the MABC-2 manual, the total test score can be converted into percentile scores to determine the level of motor development delay in children Henderson et al. (2007).

Based on normative percentile tables, the “traffic light” scoring system is employed to evaluate motor competence levels. Specifically, scores at or below the 5th percentile (red zone) indicate significant motor development difficulties. Scores between the 6th and 15th percentiles (amber zone) suggest a potential risk of motor development challenges, while scores above the 15th percentile (green zone) denote no detected motor difficulties.

The validity of the MABC-2 as a tool for assessing motor development in Chinese children has been substantiated by Hua et al. (2012), with an average content validity index of 0.985. This validation emphasizes the tool’s broad applicability across various regions within China, reflecting its comprehensive suitability for evaluating motor development abilities in Chinese children. Furthermore, the MABC-2 has been recognized for its reliability and validity in assessing FMS not only in typically developing children but also in children with ASD. Studies like Brown and Lalor (2009) support the MABC-2’s effectiveness in a universal context, further confirming its utility in diverse child populations.

#### 2.2.2 Assessment of symptom severity

This study employed the third edition of the Psycho-Educational Profile Revised in Chinese (C-PEP3) to measure the developmental levels and assess the symptom severity of children with ASD. The Psycho-Educational Profile, originally developed by Schopler & Reichler in the United States, has been adapted into its third Chinese edition through collaborative efforts by educational and medical professionals from Liaoning Normal University and Beijing Medical University (Yu, 2001).

The C-PEP 3 consists of two main sections: the Pathology Profile and the Developmental Functions Profile. The Pathology Profile is comprised of 44 items spanning five domains: affect, interpersonal relations, preferences for objects, sensory modalities, and language. Scoring codes are designated as “Absent (A),” “Mild (M),” and “Severe (S)” across these categories. The Developmental Functions Profile includes 95 items covering seven areas: imitation, perception, fine motor skills, gross motor skills, eye-hand coordination, cognitive performance, and verbal cognitive skills, with scoring codes of “Pass (P),” “Emerging (E),” and “Fail (F).” Unlike previous developmental scales, the C-PEP 3 is tailored specifically for children with ASD, capable of not only measuring the degree of impairment but also highlighting the child’s strengths and weaknesses; it also provides a foundation for educators to develop individualized education plans (Wang and Li, 2018).

Assessors are required to complete a training course certified by the Autism Rehabilitation Professional Committee of the China Association of Persons with Disabilities and must possess a C-PEP 3 assessment certification to conduct evaluations. In this study, the C-PEP 3 scores of children participating in experimental interventions were provided by cooperating ASD rehabilitation institutions, which are authorized and qualified to conduct C-PEP 3 assessments. All participants’ C-PEP 3 evaluations were conducted 1 month prior to the start of the experimental interventions.

### 2.3 Procedure

In addressing the unique challenges of assessing motor skills in children with ASD, our study adopted a holistic approach that included a comprehensive acclimation process to familiarize participants with the research setting and minimize potential discomfort. This process was vital in ensuring the children’s comfort and cooperation during the assessment, thereby enhancing the reliability of our findings. The initial phase of the study involved a month-long acclimation period at the testing institution, where the lead examiner and research assistants conducted weekly volunteer service activities, including interactive games, storytelling sessions, and art projects, to engage both the teaching staff and the children with ASD. These activities were designed to build rapport with the participants and acclimate them to the research team’s presence, fostering a cooperative atmosphere conducive to assessment. The familiarization period consisted of four sessions, each lasting approximately 2 h, conducted weekly over a month, which was especially important for the autistic group to minimize any discomfort or unfamiliarity with the examiner and the testing environment.

Subsequently, assessments were performed in a quiet movement room, specifically arranged to minimize external distractions and enhance focus. This setting proved crucial in allowing the children to focus on the tasks without undue stress or agitation. The testing phase saw the lead examiner personally accompanying the children to the testing site, where a dedicated period was allocated for the examiner to interact with each child, especially important for the autistic group to alleviate any anxiety or discomfort associated with the examiner and the unfamiliar testing environment. In the assessment, children with ASD typically required 30–40 min to complete, compared to 20–30 min for neurotypical peers, due to a combination of factors: interest-driven distractions toward colorful assessment tools, repetitive behaviors that diverted focus from task timing, the need for additional reinforcement to maintain engagement, and lower physical fitness levels affecting task completion speed. These challenges highlight the importance of adapting assessment strategies to accommodate the unique needs of children with ASD.

To ensure the accuracy and reliability of the MABC-2 translations and scoring, formal permission was obtained to create a Chinese version of the MABC-2. The translation process was carried out independently by two authors of our study, both holding doctoral degrees in sports science and experienced in early childhood movement assessment. A comprehensive training session, including theoretical and practical training and an assessor consistency check, was conducted for the principal assessor and research team members, facilitated by experts in the MABC-2 test from the United States. Through these meticulously planned procedures and validations, we achieved an inter-rater reliability score of 0.99 for the MABC-2 tests, demonstrating the effectiveness of our methodology in accurately assessing motor skills in children with ASD.

### 2.4 Data analysis

After data collection, the researcher conducted data processing. The MABC-2 test scores were divided into raw scores, standard scores, and percentile scores. In this study, all test scores were analyzed using percentile scores and standard scores. All data analyses were performed using IBM SPSS Statistics 22.0. Group differences on MABC-2 percentile scores were analyzed using descriptive data and Mann Whitney U test. Results were considered significant if *p* values were less than 0.05 and effect sizes (ES) were determined for practical significance using Cohen’s d (Cohen, 1988).

## 3 Results

### 3.1 Descriptive statistics of percentile scores for FMS

In the present study, the MABC-2 traffic light scoring system was employed to assess and distinguish the FMS of children with ASD as compared to their typically developing counterparts. The comprehensive results are tabulated in Table 1. Within the cohort of 108 typically developing children who underwent the MABC-2 assessment, 100% scored within the green zone (percentile >15%), thereby indicating an absence of motor development impairments or associated risks. Conversely, in the ASD cohort comprising 108 children, 86 (79.62%) were categorized in the red zone, signifying substantial motor development deficits. Additionally, 4 children (3.70%) were placed in the amber zone, suggestive of potential motor development risks, while only 18 children (16.67%) scored in the green zone, showing no evident motor development issues or delays. Upon comparing the two cohorts, it was found that static and dynamic balance abilities were the areas of highest proficiency, thus termed as the ‘strongest’ performance areas in the MABC-2 subtests for both groups. Manual dexterity was identified as a moderate performing area, ranking second in proficiency. In contrast, aiming and catching abilities emerged as the areas where both groups faced the most challenges, hence classified as the ‘weakest’ performance areas.

**Table 1:** Motor impairment characteristics overall and by groups.

Group	MABC-2	Red zone	Amber zone	Green zone
Children with ASD (*n* = 108)	Manual dexterity	79 (73.2%)	9 (8.3%)	20 (18.5%)
	Aiming and catching	81 (75.0%)	4 (3.7%)	23 (21.3%)
	Static and dynamic balance	67 (62.0%)	5 (4.6%)	36 (33.4%)
	Overall motor skills	86 (79.6%)	4 (3.7%)	18 (16.7%)
Typical developing children (*n* = 108)	Manual dexterity	2 (1.85%)	2 (1.85%)	104 (96.3%)
	Aiming and catching	11 (10.2%)	7 (6.5%)	90 (83.3%)
	Static and dynamic balance	1 (0.9%)	0 (0%)	107 (99.1%)
	Overall motor skills	0 (0%)	0 (0%)	108 (100%)

MABC-2 = Movement Assessment Battery for Children-2; Red Zone = significant movement difficulty; Amber Zone = the “at risk” of having a movement difficulty; Green Zone = no movement difficulty detected.

### 3.2 Comparative analysis of FMS standard scores across two child cohorts

Table 2 delineates the mean and standard deviation values for the three subtests of the MABC-2, as well as the overall motor ability standard scores, for both cohorts of children. The MABC-2, designed for children aged 3–16, has a standard score range of 1–19. It was anticipated that the cohort of typically developing children of the same age would register higher standard scores.

**Table 2:** Comparative analysis of standard scores for FMS between variables overall and by groups (mean ± standard deviation).

Category	Children with ASD (*n* = 108)	Typically developing children (*n* = 108)	Z-score	*p*-value
Manual dexterity	4.04 ± 3.05	11.04 ± 2.85	−11.32	0.000***
Aiming and catching	4.44 ± 2.77	9.20 ± 2.93	−9.49	0.000***
Static and dynamic balance	5.12 ± 4.62	11.28 ± 2.47	−9.62	0.000***
Overall motor skills	3.56 ± 3.13	10.66 ± 2.21	−11.47	0.000***

*p < 0.05, **p < 0.01, ***p < 0.001.

Our findings indicate a ceiling effect in the typically developing cohort, with 12.96% (*n* = 14), 4.63% (*n* = 5), 10.12% (*n* = 11), and 4.63% (*n* = 5) of participants scoring close to the maximum standard score of 19 across the subtests of manual dexterity, aiming and catching, and static and dynamic balance, as well as in overall motor skills. Conversely, a floor effect was observed in the cohort of children with ASD, where 12.96% (*n* = 14), 9.26% (*n* = 10), 34.26% (*n* = 37), and 38.89% (*n* = 42) scored the minimum standard score of 1 in these respective areas. Given that these extreme scores violate the assumptions of normal distribution, parametric analyses were deemed inappropriate. Consequently, the Mann–Whitney U test was employed to assess between-group differences in FMS.

The results revealed highly significant differences (*p* < 0.001) in overall motor ability scores and in the standard scores across all subtests between the two cohorts. Specifically, the FMS scores for children with ASD were significantly lower than those for typically developing children of the same age. In terms of mean scores, the autistic cohort achieved approximately half the standard scores compared to their typically developing counterparts across all evaluated domains, suggesting that their FMS were markedly compromised.

### 3.3 Gender differences in FMS between the two groups of children

A Mann–Whitney U test was conducted to analyze gender differences in FMS between the two groups of children. As shown in Table 3, the results revealed no significant gender differences in manual dexterity, aiming and grasping, static and dynamic balance, or overall motor capability (*p* > 0.05).

**Table 3:** Gender differences in fundamental movement skills between children with ASD and typically developing children (M ± SD).

Category	Children with ASD				Typically developing children			
	Male (*N* = 86)	Female (*N* = 22)	Z	p	Male (*N* = 86)	Female (*N* = 22)	Z	p
Manual dexterity	3.95 ± 3.04	4.36 ± 3.14	−0.603	0.547	11.27 ± 2.81	10.64 ± 2.56	−1.340	0.180
Aiming and catching	4.60 ± 2.76	3.82 ± 2.81	−1.275	0.202	9.31 ± 2.81	9.00 ± 3.10	−0.49	0.619
Static and dynamic balance	5.38 ± 4.90	4.09 ± 3.16	−0.755	0.450	11.31 ± 2.34	11.50 ± 2.74	−0.382	0.703
Overall motor skills	3.64 ± 3.28	3.23 ± 2.52	−0.012	0.991	10.81 ± 2.17	10.45 ± 1.92	−0.548	0.583

## 4 Discussion

The aim of this study was to investigate the performance of FMS in children with ASD and the differences compared to typically developing children of the same age, in order to better understand the motor development difficulties associated with ASD. Analysis of the results from the MABC-2 Traffic Light Scoring System revealed that approximately 80% of children with ASD obtained scores in the red or amber zone, indicating significant motor development difficulties or a risk of motor development difficulties. It is important to note that not all children diagnosed with ASD exhibit motor difficulties.

The findings of this study are consistent with previous reports on the prevalence of motor development difficulties in children with ASD. Reported that out of 101 children with ASD in England who underwent MABC assessment, 79.2% exhibited clear motor development difficulties, with an additional 9.9% at risk for motor development difficulties. Liu and Breslin (2013) compared 30 children with ASD and 30 age-matched typically developing children using the MABC and found that all typically developing children did not have motor development difficulties, while 77% of children with ASD had clear motor development difficulties and 3% were at risk for motor development difficulties. Children with ASD showed delayed development in FMS compared to typically developing children. These results have significant implications for educators, therapists, and clinicians working with ASD, particularly in designing appropriate intervention programs to effectively address motor development difficulties in children with ASD.

The findings of this study align with previous research that utilized the MABC tool for assessing and comparing children with ASD and typically developing children (Hilton et al., 2007; Quintas et al., 2018; Hu et al., 2021), although there were variations in study design and measurement scope. Conducted a large-scale screening of 101 children with ASD to assess their motor abilities, focusing on an age range of 10–14 years without including other age groups. Quintas et al. (2018) tested 14 children with ASD and 14 typically developing children using the MABC-2, with a small sample size and 12 children with ASD aged over 10, leaving only 2 children below 10 years old. Hilton et al. (2007) conducted a study with a significant sample size (*N* = 56), recognizing that the reliance on parent-reported diagnoses necessitates a consideration of the evolving diagnostic criteria within the ASD spectrum, including the reclassification of Asperger syndrome. Apart from Liu and Breslin’s study (Liu and Breslin, 2013) that compared 30 children with ASD and 30 age-matched typically developing children, other studies had small sample sizes, such as Quintas et al. (2018) (*N* = 28) and Berkeley et al. (2001) (*N* = 15). Through extensive testing and comparative analysis, this study revealed delays in motor development among children with ASD during the 7–10 year age period, indicating that motor development delay may serve as a potential window for understanding the core features of ASD (Leary and Hill, 1996).

Furthermore, the study found no gender differences in the MABC-2 assessment for both children with ASD and typically developing children, consistent with earlier findings by Henderson et al. (2007). A recent systematic review by Rodrigues et al. (2019) on MABC-2 assessment in typically developing children indicated that approximately 75% of studies showed that boys outperformed girls in gross motor skills such as aiming and catching, while approximately 65% of studies found that girls outperformed boys in fine motor skills such as manual dexterity, with no consensus reached regarding balance skills (static and dynamic balance). However, due to the exclusion of certain age groups (3–16 years) in some studies or the utilization of different versions of the MABC, gender differences may have been underestimated or overestimated. Currently, no research on gender differences in MABC testing for children with ASD has been found. In this study, starting with the assessment of FMS in children with ASD, and conducting paired comparisons with typically developing children, no gender differences were observed. This may be attributed to the imbalanced gender ratio in the study, and future research is encouraged to include a larger number of girls when assessing motor skill development in the ASD population.

In the realm of practical application, it was observed that the majority of children diagnosed with ASD successfully completed the MABC-2 test. However, their subpar scores underscored a pronounced delay and deficiency in the development of FMS. Within the MABC-2 assessment, the average scores of children with ASD in areas such as manual dexterity, aiming and catching, and both static and dynamic balance were found to be approximately half of those of their typically developing peers. This suggests that their FMS are only at par with half the proficiency level of age-matched neurotypical children. Staples and Reid (2010) conducted a study where they compared a group of children with ASD to a control group of children with comparable motor skills. The results indicated that the motor performance of the ASD group was akin to children half their age. Collectively, whether evaluated through age-matched or motor skills-matched comparisons, children with ASD consistently demonstrated a significant lag in FMS relative to their neurotypical counterparts.

Utilizing a detailed analytical method to dissect individual performance scores on the MABC-2 offers a nuanced understanding of motor skill development in children with ASD, instrumental in uncovering the nuanced underpinnings of motor challenges unique to this population. Our analysis reveals a hierarchical proficiency across its subtests, with static and dynamic balance identified as areas of relative strength, showcasing the highest levels of performance. This contrasts with the marked difficulties in tasks requiring fine motor control and coordination, such as aiming and catching, pinpointed as the least proficient areas. These findings align with research by Whyatt and Craig (2012) and MacDonald et al. (2013), highlighting the complex interplay of motor skills in ASD. The proficiency in balance tasks versus the challenges in hand-eye coordination, timing, distance judgment, and force control in aiming and catching tasks elucidate the specific motor skill challenges faced by children with ASD. This discrepancy underscores the necessity for targeted interventions aimed at enhancing fine motor skills, particularly in activities that demand high levels of coordination and control, highlighting the importance of addressing motor development in early intervention treatments.

Recent investigations further delineate the critical role of sensory integration in mitigating balance and motor control difficulties in children with ASD, with studies such as those by Oster and Zhou (2022) and Abdel Ghafar et al. (2022) illuminating profound balance and motor skill impairments, specifically highlighting how disruptions in vestibular and proprioceptive inputs significantly contribute to these challenges. These studies advocate for the utilization of balance and vestibular assessments as integral components of early intervention strategies. Complementing these findings, Sahid et al. (2019) validate the efficacy of sensory integration therapy in ameliorating sensory and motor discrepancies, demonstrating specific improvements in tasks requiring coordination and balance. The seamless integration of inputs from the vestibular, somatosensory, and visual systems emerges as indispensable for postural stability, especially evident in activities requiring static balance. The elucidation of the intricate relationship between sensory processing disruptions and motor control difficulties in ASD accentuates the necessity for interventions aimed at bolstering sensory integration, thereby promising substantial enhancements in balance and motor proficiency, and by extension, the overall quality of life for affected individuals.

Furthermore, the study identified consistent deficits or inadequacies in certain skill operations among children with ASD, such as timed threading boards, timed placement of small mushrooms, and single-leg standing. These test tasks are inherently complex, with scores being determined based on the accuracy of the operation and age-related time constraints. The findings revealed that children with ASD performed notably worse in the timed tasks of the MABC-2 test compared to their neurotypical peers. Children with ASD might face unique challenges in these areas, leading to an inevitable trade-off: maintaining a deliberately slow pace to achieve higher accuracy. Moreover, it’s plausible that children with ASD might not fully grasp the concept of time or the principle of maximizing duration during the test, or they might be indifferent to the test’s requirements. This suggests potential difficulties faced by children with ASD when executing complex tasks. It is recommended that further research be conducted to delve into the time perception and complex task execution abilities of children with ASD.

This study’s limitations underscore the necessity for a more expansive approach in future research. Firstly, the relatively modest sample, confined to children within China, calls for the inclusion of diverse international populations in subsequent studies to enhance the outcomes’ robustness and external validity. Moreover, the omission of demographic data such as socioeconomic status and parental educational levels represents a significant gap, given their potential impact on developmental trajectories in children with ASD. Future investigations should aim to incorporate these critical demographic variables, facilitating a deeper exploration of the interplay between familial backgrounds and motor development. Additionally, it is recommended that future research adopts longitudinal study designs to include comprehensive assessments of intelligence alongside motor abilities, aiming to illuminate the complex mechanisms behind the observed developmental delays and challenges in children with ASD. Such an approach will directly address the interplay between motor skills and intellectual levels, providing a more nuanced understanding of ASD’s developmental implications.

## 5 Conclusion

Children with ASD manifest a significant delay in the development of FMS compared to their typically developing age-matched peers. In the MABC-2 test, whether considering scores in manual dexterity, aiming and catching, static and dynamic balance, or overall motor skills, children with ASD consistently scored markedly lower than their neurotypical counterparts, with no discernible gender differences. While motor skills are not traditionally regarded as a primary diagnostic category for ASD, our findings indicate that approximately 80% of children with ASD either experience motor developmental challenges or are at risk for such challenges.

This study further underscores that motor developmental challenges are a core characteristic of ASD. It is recommended that clinicians, when diagnosing and treating children with ASD, should not only consider the developmental domains described in traditional ASD diagnostic manuals but should also assess for the presence of motor developmental challenges. Evidence-based interventions should be implemented for children who are diagnosed with both ASD and motor developmental deficiencies. Furthermore, conducting longitudinal research during critical periods of motor skill development in children with ASD may provide a deeper understanding of their motor trajectories and developmental pace and might offer insights into contributory factors.

## Data availability statement

The original contributions presented in the study are included in the article/supplementary material, further inquiries can be directed to the corresponding author.

## Ethics statement

The studies involving humans were approved by China University of Geosciences (Wuhan). The studies were conducted in accordance with the local legislation and institutional requirements. Written informed consent for participation in this study was provided by the participants’ legal guardians/next of kin.

## Author contributions

LD: Data curation, Funding acquisition, Methodology, Writing – original draft, Writing – review & editing. RF: Supervision, Conceptualization, Project administration, Writing – review & editing. BS: Project administration, Resources, Writing – review & editing. JB: Project administration, Resources, Writing – review & editing. YP: Data curation, Investigation, Supervision, Validation, Visualization, Writing – review & editing. YS: Data curation, Investigation, Validation, Visualization, Writing – review & editing.
